# Anatomy of Omicron BA.1 and BA.2 neutralizing antibodies in COVID-19 mRNA vaccinees

**DOI:** 10.1038/s41467-022-31115-8

**Published:** 2022-06-13

**Authors:** Emanuele Andreano, Ida Paciello, Silvia Marchese, Lorena Donnici, Giulio Pierleoni, Giulia Piccini, Noemi Manganaro, Elisa Pantano, Valentina Abbiento, Piero Pileri, Linda Benincasa, Ginevra Giglioli, Margherita Leonardi, Piet Maes, Concetta De Santi, Claudia Sala, Emanuele Montomoli, Raffaele De Francesco, Rino Rappuoli

**Affiliations:** 1 Monoclonal Antibody Discovery (MAD) Lab, Fondazione Toscana Life Sciences, Siena, Italy; 2 Department of Pharmacological and Biomolecular Sciences DiSFeB, University of Milan, Milan, Italy; 3 INGM, Istituto Nazionale Genetica Molecolare “Romeo ed Enrica Invernizzi”, Milan, Italy; 4 VisMederi S.r.l, Siena, Italy; 5 VisMederi Research S.r.l., Siena, Italy; 6 KU Leuven, Rega Institute, Department of Microbiology, Immunology and Transplantation, Laboratory of Clinical and Epidemiological Virology, Leuven, Belgium; 7 Department of Molecular and Developmental Medicine, University of Siena, Siena, Italy; 8 Department of Biotechnology, Chemistry and Pharmacy, University of Siena, Siena, Italy

**Keywords:** Antibodies, VDJ recombination, RNA vaccines, SARS-CoV-2

## Abstract

SARS-CoV-2 vaccines, administered to billions of people worldwide, mitigate the effects of the COVID-19 pandemic, however little is known about the molecular basis of antibody cross-protection to emerging variants, such as Omicron BA.1, its sublineage BA.2, and other coronaviruses. To answer this question, 276 neutralizing monoclonal antibodies (nAbs), previously isolated from seronegative and seropositive donors vaccinated with BNT162b2 mRNA vaccine, were tested for neutralization against the Omicron BA.1 and BA.2 variants, and SARS-CoV-1 virus. Only 14.2, 19.9 and 4.0% of tested antibodies neutralize BA.1, BA.2, and SARS-CoV-1 respectively. These nAbs recognize mainly the SARS-CoV-2 receptor binding domain (RBD) and target Class 3 and Class 4 epitope regions on the SARS-CoV-2 spike protein. Interestingly, around 50% of BA.2 nAbs did not neutralize BA.1 and among these, several targeted the NTD. Cross-protective antibodies derive from a variety of germlines, the most frequents of which were the IGHV1-58;IGHJ3-1, IGHV2-5;IGHJ4-1 and IGHV1-69;IGHV4-1. Only 15.6, 20.3 and 7.8% of predominant gene-derived nAbs elicited against the original Wuhan virus cross-neutralize Omicron BA.1, BA.2 and SARS-CoV-1 respectively. Our data provide evidence, at molecular level, of the presence of cross-neutralizing antibodies induced by vaccination and map conserved epitopes on the S protein that can inform vaccine design.

## Abstract (web summary)

Here, Andreano and Paciello et al. show, at single cell level, the functional and genetic characteristics underlying the Omicron BA.1 and BA.2 cross-protective antibody response in naïve and previously infected COVID-19 vaccinees.

## Introduction

Since its first appearance in December 2019, more than 495 million cases of SARS-CoV-2 infections were reported worldwide, with over 6.1 million deaths. Effective vaccines against the virus that first appeared in Wuhan, China, have been developed with unprecedented speed. However, their ability to contain the global pandemic has been compromised by the inability to timely deliver vaccines to low-income countries and by the appearance of several antigenic variants which escaped the natural and vaccine-induced immunity^1–3^. The main variants that emerged so far, and are listed as variants of concern (VoCs), are named B.1.1.7 (Alpha), B.1.351 (Beta), P.1 (Gamma), B.1.617.2 (Delta), and B.1.1.529.1 (Omicron; BA.1)^4,5^. The latter one showed to be the most efficient in spreading into partially immune populations and in a few months from its appearance has conquered most regions of the world^6,7^. Shortly after the appearance of the Omicron variant BA.1, the sublineage BA.2 (B.1.1.529.2) was identified, and it is now replacing the initial BA.1 variant worldwide^8,9^. Previous reports have shown that the unprecedented number of mutations carried on the Omicron BA.1 and BA.2 S protein drastically reduce the neutralizing efficacy of sera from infected and vaccinated people and that this VoC can escape more than 85% of nAbs described in literature, including several antibodies approved for clinical use by regulatory agencies^10–18^. Despite these observations, recent reports have shown different profiles of immune evasion between omicron BA.1 and BA.2^19–21^. While serum activity and neutralization efficacy of selected monoclonal antibodies against Omicron BA.1 and BA.2 have been reported, the functional and genetic anatomy of nAbs elicited in naïve (seronegative) and convalescent (seropositive) people immunized with two doses of the BNT162b2 mRNA vaccine remains to be explored. Taking advantage of our previous work^22^, we tested 276 human monoclonal antibodies able to neutralize the original SARS-CoV-2 virus isolated in Wuhan, for their ability to neutralize the Omicron BA.1 and BA.2 variants, and the distantly related SARS-CoV-1 virus. Our work unravels the genetic signature of cross-protective antibodies and mapped conserved sites of pathogen vulnerability on the S protein that can be used to design the next generation of sarbecovirus vaccines.

## Results

### Distribution of BA.2 and BA.2 mutations on immunodominant sites

The SARS-CoV-2 B.1.1.529.1 (BA.1) and B.1.1.529.2 (BA.2) Omicron variants harbor 37 and 31 mutated residues in the spike (S) glycoprotein respectively (Supplementary Fig. 1a). The receptor binding domain (RBD) and N terminal domain (NTD) immunodominant sites are both highly mutated^14,19^. The NTDs of BA.1 and BA.2 carry 11 and 7 mutations respectively. BA.1 shows to be more remodeled compared to BA.2, presenting three substitutions (27%), A67V, T95I, and G142D, 5 deleted residues (46%), Δ69–70 and Δ143–145, and three inserted residues (27%), ins214EPE. On the other hand, BA.2 presents a NTD more similar to the original Wuhan virus carrying only four substitutions (57%), T19I, A27S, G142D, and V213G, and three deleted residues (43%), Δ24–26. The RBDs of BA.1 and BA.2 show a lower degree of plasticity compared to NTD, as only substituted residues are present in this domain. Within the RBD, which contains 15 and 16 mutations in total, the receptor binding motif (RBM), spanning from residues S438 to Y508^23^, is the most mutated region. In fact, BA.1 RBM contains 10/15 (67%) mutated residues, while BA.2 carries 8/16 (50%) mutations. The eight mutated residues in the RBM of BA.2 are all shared with BA.1. Mutated residues in the RBM overlap with the epitopes of Class 1 and Class 2 neutralizing antibodies (nAbs), like J08^24,25^, that target epitopes spanning from the left shoulder, through the neck and upper part of the right shoulder of the S protein (Supplementary Fig. 1b–d)^26,27^. Class 3 and 4 clusters of antibodies target the lower portion of the RBD, and their epitopes are located on the right and left flanks of this domain. Class 3 nAbs, like S309^28^, target the right flank of the RBD where only 2/15 (13%) and 2/16 (12%) of all BA.1 and BA.2 mutations are found respectively (Supplementary Fig. 1d). Class 4 mAbs, like CR3022^29^, are directed towards the left flank of the RBD which shows 3/15 (20%) and 6/16 (37%) mutations for BA.1 and BA.2 respectively (Supplementary Fig. 1b).

### Omicron effects on vaccine-induced nAbs

**Fig. 1:**
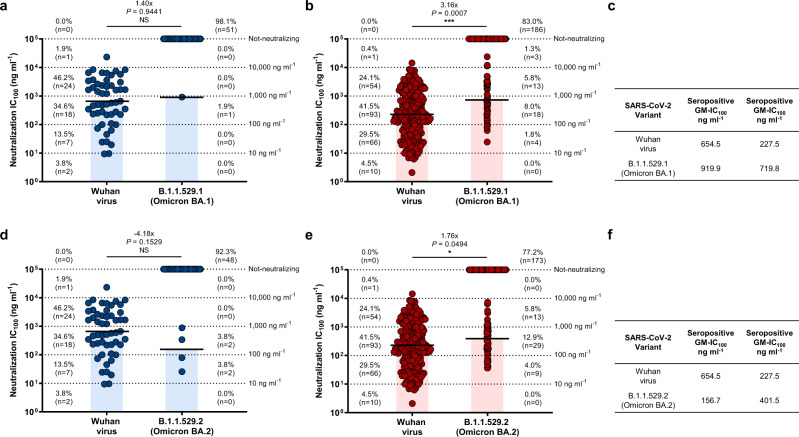
Functional characterization of Omicron BA.1 and BA.2 nAbs. **a**–**f** Dot charts show the neutralization potency, reported as IC_100_ (ng ml^−1^), of nAbs tested against the original SARS-CoV-2 virus first detected in Wuhan, the BA.1 and BA.2 Omicron VoC for seronegatives (**a**, **c**, **d**, **f**) and seropositives (**b**, **c**, **e**, **f**). The number and percentage of nAbs from individuals who were seronegative versus seropositive, fold change, neutralization IC_100_ geometric mean (black lines, blue and red bars) and statistical significance are denoted on each graph. Technical duplicates were performed for each experiment. A non-parametric Mann–Whitney *t*-test was used to evaluate statistical significances between groups. Two-tailed *P* value significances are shown as *P < 0.05, **P < 0.01, ***P < 0.001. NS, not significant. **c**, **f** Tables show the IC_100_ geometric mean (GM) of all nAbs pulled together from each group against Wuhan, BA.1 (**c**) and BA.2 (**f**) viruses.

**Fig. 2:**
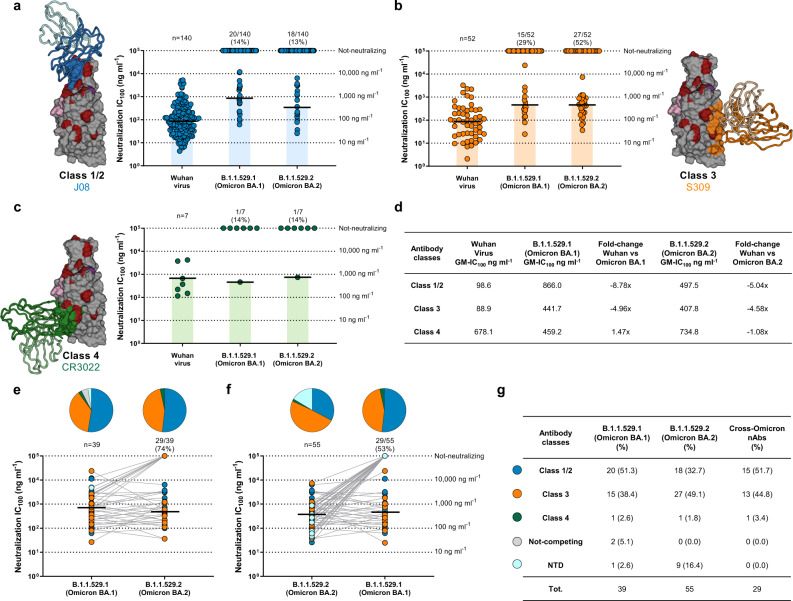
Distribution of Omicron BA.1 and BA.2 nAbs. **a**–**c** Dot charts show the distribution of Class 1/2 (**a**), Class 3 (**b**) and Class 4 (**c**) nAbs against the original SARS-CoV-2 virus first detected in Wuhan and the BA.1 and BA.2 Omicron VoCs. The number and percentage of nAbs and neutralization IC_100_ geometric mean (black lines, light blue, orange, and green bars) are denoted on each graph. **d** The table summarizes the IC_100_ geometric mean of nAbs against Wuhan, BA.1 and BA.2 VoCs and the GM-IC_100_ fold-change between the Omicron variants and the ancestral Wuhan virus. **e**, **f** Dot charts show the neutralization potency, reported as IC_100_ (ng ml^−1^), of nAbs against Omicron BA.1 and their ability to cross-neutralize BA.2 (**e**) and of nAbs against Omicron BA.2 and their ability to cross-neutralize BA.1 (**f**). The number and percentage of nAbs and neutralization IC_100_ geometric mean (black lines) are denoted on each graph. Pie charts show the distribution of nAbs based on their ability to bind Class 1/2 (blue), Class 3 (orange), and Class 4 (dark green) regions on the RBD, as well as not-competing nAbs (gray) and NTD-targeting nAbs (cyan). **g** The table summarizes number and percentages of Class1/2 (blue), Class 3 (orange), Class 4 (dark green) not-competing (gray) and NTD-targeting nAbs (cyan) for BA.1, BA.2, and cross-Omicron nAbs.

To understand the impact of SARS-CoV-2 Omicron on the antibody response, we evaluated the neutralization activity of 276 nAbs previously isolated from seronegative (*n* = 52) and seropositive (*n* = 224) donors immunized with the BNT162b2 mRNA vaccine (Fig. 1)^22^. Data obtained from the neutralization analyses against Omicron BA.1 and BA.2 were compared with the neutralization data against the original Wuhan virus analyzed in our previous work^22^. While BA.1 cross-neutralizing nAbs were identified in all seropositives, these antibodies were found only in one out of five seronegatives (20%). Conversely, BA.2 cross-neutralizing nAbs were identified in all seropositives and in three out of five seronegatives (60%) (Supplementary Table 1). Only 1/52 (1.9%) and 4/52 (7.7%) nAbs from seronegatives neutralized Omicron BA.1 and BA.2 respectively, with a medium-low neutralization potency (Fig. 1a, c, d, f). As for seropositive vaccinees, 38/224 (17.0%) BA.1 and 51/224 (22.8%) BA.2 nAbs were identified (Fig. 1b, c, e, f). BA.1 nAbs showed a 3.16-fold decreased neutralization potency compared to the Wuhan virus, while BA.2 nAbs showed only a 1.76-fold decrease (Fig. 1b, c, e, f). Overall, 39 and 55 nAbs against BA.1 and BA.2 were identified respectively and none of these antibodies showed an extreme neutralization potency (IC_100_ below 10 ng ml^−1^). To identify immunodominant sites of neutralization against BA.1 and BA.2, a flow cytometry-based competition assay was performed. In our previous study, we found that 215/276 (77.9%; 37 from seronegatives and 178 from seropositives) nAbs bound to the S protein RBD and the majority of these antibodies were competing with J08, which epitope spans between Class 1 and Class 2 regions, and S309, which target the Class 3 region^22^. In this work, all 215 RBD targeting-nAbs were additionally tested by competition assay against Class 4 targeting mAb CR3022. While Class 1/2, Class 3 and Not-competing nAbs were found in both seronegatives and seropositives, Class 4 competing nAbs were found exclusively in seropositives (Supplementary Fig. 2a; Supplementary Table 2). In both groups, Class 1/2 competing nAbs was the most abundant (70.3% in seronegatives and 64.0% in seropositives), followed by Not-competing (10.8%) and Class 3-competing nAbs (27.0%) for seronegatives and seropositives respectively. When tested for their neutralization activity against the BA.1 and BA.2 omicron variants, we observed that RBD-targeting Class 3 directed nAbs are the most abundant and potent class of nAbs against both BA.1 (29%) and BA.2 (52%) (Fig. 2b–d). In terms of overall neutralization potency, GM-IC_100_, BA.1 nAbs showed a higher loss compared to BA.2 nAbs (Fig. 2d). Following, we evaluated the binding distribution of the 39 BA.1 and 55 BA.2 neutralizing antibodies and investigated the class of nAbs able to cross-neutralize both Omicron variants. Of the 39 BA.1 nAbs, the majority recognized the Class 1/2 region (*n* = 20; 51.3%), followed by Class 3 (*n* = 15; 38.5%), while nAbs targeting Class 4 region (*n* = 1; 2.6%), Not-competing (*n* = 2; 5.1%) and targeting the NTD (*n* = 1; 2.6%) were the least represented (Fig. 2e–g). As for the 55 BA.2 nAbs, the majority recognized the RBD Class 3 region (*n* = 27; 49.1%), followed by Class 1/2 (n = 18; 32.7%), NTD-targeting nAbs (*n* = 9; 16.4%), and Class 4 nAbs (1.8%) (Fig. 2e–g). Cross-Omicron variants nAbs were also characterized. While up to 74% (*n* = 29/39) of BA.1 nAbs were able to neutralize BA.2, only 53% (*n* = 29/55) of BA.2 nAbs showed cross-functional activity against BA.1. The majority of cross-Omicron nAbs were represented by Class 1/2 targeting nAbs (*n* = 15; 51.7%), followed by Class 3 (*n* = 13; 44.8%) and Class 4 (*n* = 1; 3.4%) targeting nAbs. None of the NTD-targeting nAbs were found to be cross-functional (Fig. 2e–g).

### Antibody cross-protection to SARS-CoV-1

We investigated the ability of the BNT162b2 COVID-19 mRNA vaccine-elicited nAbs to neutralize the distantly related SARS-CoV-1 (SARS1) virus, using a lentiviral vector derived pseudoparticles, to identify immunodominant sites of cross-protection to different coronaviruses^30^. All 276 previously identified nAbs were tested for their ability to bind the SARS1 S protein (Supplementary Fig. 2b, c). Four out of five seropositives (80%) and one out of five seronegatives (20%) showed nAbs able to bind the SARS1 S protein (Supplementary Fig. 3a; Supplementary Table 1). Of the 52 nAbs isolated from seronegatives 3 (5.8%) recognized SARS1 S protein, while 16 out of 224 nAbs (7.1%) from seropositives were able to bind this antigen (Supplementary Fig. 3a; Supplementary Table 1). When nAbs were tested for their neutralization activity against SARS1, only 1 (1.9%) and 10 (4.5%) nAbs were found from seronegatives and seropositives respectively (Supplementary Fig. 3b; Supplementary Table 1). All SARS1 cross-neutralizing nAbs recognized the SARS-CoV-2 S protein RBD. Class 4 targeting nAbs able to cross-neutralize SARS1 were overall the most frequent, followed by Class 3 binding nAbs (Supplementary Fig. 3c; Supplementary Table 1). While Class 4 was the most frequent, Class 3 targeting nAbs showed the highest neutralization potency (Supplementary Fig. 3c, d; Supplementary Table 1). None of the 140 Class 1/2 regions targeting nAbs were able to cross-neutralize SARS1. Among the 11 SARS1 cross-neutralizing nAbs, the majority recognized the Class 3 region (*n* = 6; 54.5%), followed by Class 4 (*n* = 4; 36.4%) and Not-competing (*n* = 1; 9.1%) nAbs (Supplementary Fig. 3c, d).

### Antibody repertoire to Omicron and SARS-CoV-1

**Fig. 3:**
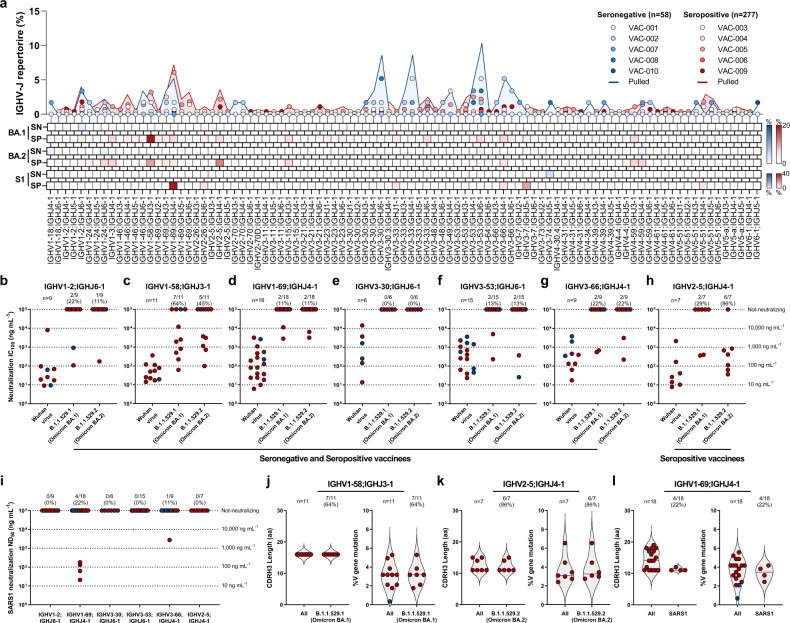
Genetic characterization of BA.1, BA.2, and SARS1 nAbs. **a** The graph shows the IGHV-J rearrangement frequencies between vaccinees who were seronegative (SN) and seropositive (SP) (top), and the frequency within BA.1 (top heatmap), BA.2 (middle heatmap), and SARS1 (S1) (bottom heatmap) nAbs. **b**–**h** The graphs show the neutralization potency (IC_100_) of IGHV1-2;IGHJ6-1 (**b**), IGHV1–58;IGHJ3-1 (**c**), IGHV1–69;IGHJ4-1 (**d**), IGHV3–30;IGHJ6-1 (**e**), IGHV3–53;IGHJ6-1 (**f**), IGHV3–66;IGHJ4-1 (**g**) and IGHV2–5;IGHJ4-1 (**h**) gene-derived nAbs, against the original SARS-CoV-2 virus first detected in Wuhan and the BA.1 and BA.2 VoCs. **i** The graph shows the neutralization potency (ND_50_) of predominant germlines against SARS1. **j**–**l** Violin plots show differences in the aminoacidic (aa) CDRH3 length and percentage of V gene somatic mutations for all IGHV1–58;IGHJ3-1 (**j**) IGHV2–5;IGHJ4-1 (**k**) and IGHV1–69;IGHJ4-1 (**l**) antibodies compared to BA.1, BA.2, and SARS1 nAbs respectively.

To investigate the genetic basis of antibody cross-protection against BA.1, BA.2, and SARS1, we interrogated the functional antibody repertoire. In our previous work, we identified five predominant germlines shared among seronegatives and seropositives (IGHV1-2;IGHJ6-1, IGHV1–69;IGHJ4-1, IGHV3–30;IGHJ6-1, IGHV3–53;IGHJ6-1 and IGHV3–66;IGHJ4-1), and one rearrangement (IGHV2–5;IGHJ4-1), that encoded for potently neutralizing antibodies able to protect against all VoC from alpha (B.1.1.7) to delta (B.1.617.2), found exclusively in seropositives (Fig. 3a, top panel)^22^. Cross-protective nAbs against BA.1, BA.2, and SARS1 use a variety of genetic rearrangements (Fig. 3a, top, middle, and bottom heatmaps). The most frequent among BA.1 nAbs was the non-predominant gene rearrangement IGHV1–58;IGHJ3-1 (64.0%; 7/11) found in both seronegatives and seropositives, while predominant gene-derived nAbs only rarely were able to neutralize BA.1 (10/64; 15.6%) (Fig. 3a, top panel, and b-h). The most abundant germline among BA.2 nAbs is the predominant IGHV2–5;IGHJ4-1 gene family (6/7; 86%), while the remaining 57 predominant gene-derived nAbs poorly neutralized this variant (7/57; 12.3%) as observed for BA.1 (Fig. 3). Interestingly, we found that IGHV1–24;IGHJ6-1 gene-derived nAbs, known to encode for NTD-targeting antibodies^31^, are abundantly used to protect from BA.2 but not from BA.1 or SARS1. As for SARS1 cross-neutralizing antibodies, only 7.8% (5/64) of the previously identified predominant germlines showed protection (Fig. 3i). Despite this observation, the most abundant germline used by these nAbs was the predominant IGHV1–69;IGHJ4-1 (4/; 22%) which was found in both seronegatives and seropositives and showed a medium-high 50% neutralization dose (ND_50_) (Fig. 3a, top panel; Fig. 3i). None of the predominant gene-derived antibodies able to neutralize BA.1 and BA.2 cross-neutralized SARS1. We further analyzed the IGHV1–58;IGHJ3-1, IGHV2–5;IGHJ4-1 and IGHV1–69;IGHJ4-1 germlines which showed to be predominant in the functional response against BA.1, BA.2 and SARS1 and observed key differences among these rearrangements. All IGHV1–58;IGHJ3-1 nAbs bound the RBD and targeted Class 1/2 region (Supplementary Fig. 4a, d), and had heavy chain complementary determining region 3 (CDRH3) length of 16 amino acid (aa) with a median V gene mutation level of 3% (Fig. 3j). All IGHV2–5;IGHJ4-1 bound the RBD and targeted the Class 3 region on the RBD, carrying mainly a CDRH3 length of 10 aa and a median V gene mutation level of 3% (Fig. 3k; Supplementary Fig. 4b, d). IGHV1–58;IGHJ3-1 and IGHV2–5;IGHJ4-1 nAbs shows similar genetic characteristics independently from their ability to neutralize BA.1 and BA.2 respectively compared to the original Wuhan virus. IGHV1–69;IGHJ4-1 derived nAbs showed to be able to recognize both NTD and RBD, and to target Class 1/2 and Class 3 regions on this latter domain (Supplementary Fig. 4c, d). Conversely, IGHV1–69;IGHJ4-1 SARS1 nAbs, showed to bind only to the RBD and to target exclusively the Class 3 epitope region (Supplementary Fig. 4c, d). IGHV1–69;IGHJ4-1 nAbs showed to use a heterogenous CDRH3 length with SARS1 nAbs spanning from 11 to 12 amino acids and V gene mutation levels averaging 3.4% (Fig. 3l).

## Discussion

In this work, we deeply characterized an extensive panel of BNT162b2 mRNA vaccine elicited-neutralizing human monoclonal antibodies against the heavily mutated Omicron variant BA.1, its sublineage BA.2, and the distantly related SARS-CoV-1. We found that only 14.2, 19.9. and 4.0% of our antibody panel was able to neutralize the Omicron BA.1, BA.2, and the SARS-CoV-1 virus respectively. Remarkably, from the group of seronegative people vaccinated with two doses of the BNT162b2 mRNA vaccine, we observed that only 5 of the 52 nAbs were able to neutralize BA.1 and BA.2, while none of their antibodies retained functionality against SARS-CoV-1. Cross-neutralizing antibodies were mainly isolated from vaccinees with previous infection, highlighting once again the broad cross-protection conferred by hybrid immunity^22,32^. Despite these results, we observed that antibody germlines mainly involved in BA.1 and SARS-CoV-1 cross-protection (IGHV1–58;IGHJ3-1 and IGHV1–69;IGHJ4-1) were used in both seronegative and seropositive vaccinees and differed from those initially identified against the original Wuhan virus and pre-omicron variants^33,34^. Interestingly, cross-protection to BA.2 was mainly driven by the Class 3 targeting germline IGHV2–5;IGHJ4-1, which was found to poorly neutralize BA.1. This scenario could partially be explained by the lack of the G446S mutation on the BA.2 spike protein, as mutations on this residue were previously shown to evade Class 3 targeting antibodies^35^. In addition to RBD-targeting antibodies, nAbs targeting the NTD “supersite” also showed to participate in the cross-protection to BA.2, as this sublineage shows a lower degree of mutations in this domain compared to BA.1. The re-acquired functionality of IGHV2–5;IGHJ4-1 Class 3 and NTD-targeting nAbs could partially explain, at the molecular level, the reason behind the higher nAbs neutralization potency to BA.2 observed in this study as well as the higher serum neutralization activity observed against BA.2 compared to BA.1 in vaccinated subjects^21^. Overall, this scenario suggests that a third booster dose in naïve people could enhance germline maturation and induce a more broad and persistent antibody response^36^. Firstly, a third booster dose could drive affinity maturation of poorly cross-reactive but predominant RBD-targeting B cell germlines elicited following SARS-CoV-2 Wuhan infection or vaccination, which were shown to persist for up to 6 months in the draining lymph nodes of vaccinated individuals^37^. Secondly, a third booster dose will also expand the population of NTD-targeting B cells, which were previously shown to be a prime target of COVID-19 mRNA vaccination^12,38^, and could therefore play a pivotal role in the cross-protection against the fast-spreading BA.2 omicron variant. In addition, these data suggest that primary immunization with two doses of vaccine in naïve people is not sufficient to elicit a meaningful proportion of cross-neutralizing antibodies and that this requires a secondary immune response that can be obtained by vaccinating previously infected people or by providing a third booster dose^39^. Further studies will be necessary to understand whether a booster dose in naïve people can elicit a hybrid immunity-like antibody response and to define the molecular basis of cross-protection in this population. In addition to previous observations on the activity of different classes of antibodies against SARS-CoV-2 and related sarbecoviruses^40–44^, we herein comprehensively defined for the first time the anatomy of BA.1, BA.2, and SARS-CoV-1 neutralizing antibodies, highlighting epitope regions and predominant germlines responsible for cross-protection to these viruses. Our data can support the design of next-generation COVID-19 vaccines broadly protective against current and future coronavirus threats.

## Methods

### Enrollment of COVID-19 vaccinees and human sample collection

Human neutralizing antibodies tested in this work were isolated from COVID-19 vaccinated donors, of both sexes (six males and four females), who gave their written consent, thanks to a collaboration with the Azienda Ospedaliera Universitaria Senese, Siena (IT). The study was approved by the Comitato Etico di Area Vasta Sud Est (CEAVSE) ethics committees (Parere 17065 in Siena) and conducted according to good clinical practice in accordance with the declaration of Helsinki (European Council 2001, US Code of Federal Regulations, ICH 1997). This study was unblinded and not randomized. No statistical methods were used to predetermine sample size.

### Transcriptionally active PCR expression of neutralizing antibodies

The transcriptionally active PCR (TAP) expression of neutralizing antibodies (nAbs) was performed as previously described^22,25^. Antibodies heavy and light chain vectors were initially digested using restriction enzymes AgeI, SalI, and Xho. PCR II products were ligated using the Gibson Assembly NEB into 25 ng of respective human Igγ1, Igκ, and Igλ expression vectors^45,46^. TAP reaction was performed using 5 μl of Q5 polymerase (NEB), 5 μl of GC Enhancer (NEB), 5 μl of 5X buffer, 10 mM of dNTPs, 0.125 μl of forward/reverse primers (forward: TTAGGCACCCCAGGCTTTAC; reverse: AGATGGTTCTTTCCGCCTCA) and 3 μl of ligation product, using the following cycles: 98 °C for 2 min, 35 cycles 98 °C for 10 s, 61 °C for 20 s, 72 °C for 1 min and 72 °C for 5 min. TAP products were purified, quantified using the Qubit Fluorometric Quantitation assay (Invitrogen), and used for transient transfection in Expi293F cell line (Thermo Fisher, Cat# A14527) following the manufacturer’s instructions.

### SARS-CoV-2 BA.1 and BA.2 authentic viruses neutralization assay

All SARS-CoV-2 authentic virus neutralization assays were performed in the biosafety level 3 (BSL3) laboratories at Toscana Life Sciences in Siena (Italy) and Vismederi Srl, Siena (Italy). BSL3 laboratories are approved by a Certified Biosafety Professional and are inspected every year by local authorities. To evaluate the neutralization activity of identified nAbs against SARS-CoV-2 and BA.1 and BA.2 Omciron VoCs a cytopathic effect-based microneutralization assay (CPE-MN) was performed^22,25^. Briefly, nAbs were co-incubated with a SARS-CoV-2 viral solution containing 100 median Tissue Culture Infectious Dose (100 TCID_50_) of virus for 1 h at 37 °C, 5% CO_2_. The mixture was then added to the wells of a 96-well plate containing a sub-confluent Vero E6 cell monolayer (ATCC, Cat# CRL-1586). Plates were incubated for 3–4 days at 37 °C in a humidified environment with 5% CO_2_, then examined for CPE by means of an inverted optical microscope by two independent operators. All nAbs were tested at a starting dilution of 1:10, diluted step 1:2, and the IC_100_ was evaluated based on their initial concentration. Technical duplicates for each experiment were performed. In each plate, positive and negative control were used as previously described^22,25^.

### SARS-CoV-2 virus variants CPE-MN neutralization assay

The SARS-CoV-2 Omicron BA.1 (B.1.1.529.1) and BA.2 (B.1.1.529.2) viruses used to perform the CPE-MN neutralization assay was supplied and sequenced by the NRC UZ/KU Leuven (Leuven, Belgium). Sequences were deposited on GISAID with the following ID: EPI_ISL_6794907 (BA.1) and EPI_ISL_10654979 (BA.2).

### SARS-CoV-2 S protein competition assay

A competitive flow cytometry-based assay was performed to characterize nAbs binding profiles to SARS-CoV-2 S-protein as previously described^22^. Briefly, magnetic beads (Dynabeads His-Tag, Invitrogen) were covered with His-tagged S-proteins, following manufacturers’ instructions. Then, 40 mg/mL of beads-bound-S-protein were incubated with unlabeled nAbs for 40 min at RT. Following incubation, samples were washed with PBS and incubated with fluorescently labeled Class 1/2 (J08-A647), Class 3 (S309-A488), or Class 4 (CR3022-A647) S-protein nAbs binders. Antibodies labeling was performed using Alexa Fluor NHS Ester kit (Thermo Scientific). Following 40 min of incubation at RT, the beads-antibodies mix was washed with PBS, resuspended in 150 μl of PBS-BSA 1%, and acquired using BD LSR II flow cytometer (Becton Dickinson). Results were analyzed using FlowJo™ Software (version 10). Beads with or without S-protein incubated with labeled antibodies were used as positive and negative controls respectively.

### SARS-CoV-1 S protein binding assay

Expi293F cells (Thermo Fisher, Cat# A14527) were transiently transfected with SARS-CoV-1 S-protein expression vectors (pcDNA3.3_CoV1_D28) using Expifectamine Enhancer according to the manufacturer’s protocol (Thermo Fisher). Two days later, to exclude dead cells from analysis, Expi293F were harvested, dispensed into a 96-well plate (3 × 10^5^ cell/well), and stained for 30 min at room temperature (RT) with Live/Dead Fixable Aqua reagent (Invitrogen; Thermo Scientific) diluted 1:500. Following Live/Dead staining, cells were washed with PBS and incubated with nAbs candidates for 40 min at RT. Next, to identify the SARS-CoV-1 S protein mAbs binders, cells were washed and stained with the Alexa Fluor 488-labeled secondary antibody Goat anti-Human IgG (H + L) secondary antibody (Invitrogen) diluted 1:500. After 40 min of incubation, labeled cells were washed, resuspended in 150 μl of PBS, and analyzed using the BD LSR II flow cytometer (Becton Dickinson). Cells incubated with the SARS-CoV-1 nAb binder (S309) or incubated only with the secondary antibody were used as positive and negative controls respectively. Data were collected with the BD FACSDiva Software v9.0 (BD Biosciences) and analyzed with FlowJo™ Software (version 10).

### HEK293TN-hACE2 cell line generation

HEK293TN-hACE2 cell line was generated by lentiviral transduction of HEK293TN (System Bioscience, Cat# LV900A-1) cells as described in Notarbartolo et al.^47^. Lentiviral vectors were produced following a standard procedure based on calcium phosphate co-transfection with 3rd generation helper and transfer plasmids. The transfer vector pLENTI_hACE2_HygR was obtained by cloning of hACE2 from pcDNA3.1-hACE2 (a gift from Fang Li, Addgene #145033) into pLenti-CMV-GFP-Hygro (a gift from Eric Campeau & Paul Kaufman, Addgene #17446). pLENTI_hACE2_HygR is now made available through Addgene (Addgene #155296). HEK293TN-hACE2 cells were maintained in DMEM, supplemented with 10% FBS, 1% glutamine, 1% penicillin/streptomycin, and 250 μg/ml Hygromycin (GIBCO).

### Production of SARS-CoV-1 pseudoparticles

SARS-CoV1 lentiviral pseudotype particles were generated as described in Conforti et al. for SARS-CoV-2^30^. SARS-CoV1 SPIKE plasmid pcDNA3.3_CoV1_D28 is a gift from a gift from David Nemazee (Addgene plasmid # 170447).

### SARS-CoV-1 neutralization assay

For neutralization assay, HEK293TN-hACE2 cells (System Bioscience, Cat# LV900A-1) were plated in white 96-well plates in a complete DMEM medium. 24 h later, cells were infected with 0.1 MOI of SARS-CoV-1 pseudoparticles that were previously incubated with serial dilution of purified or not purified (cell supernatant) mAb. In particular, a 7-point dose-response curve (plus PBS as untreated control), was obtained by diluting mAb or supernatant respectively five-fold and three-fold. Thereafter, nAbs of each dose-response curve point was added to the medium containing SARS-CoV-1 pseudoparticles adjusted to contain 0.1 MOI. After incubation for 1 h at 37 °C, 50 µl of mAb/SARS-CoV-1 pseudoparticles mixture was added to each well and plates were incubated for 24 h at 37 °C. Each point was assayed in technical triplicates. After 24 h of incubation cell infection was measured by luciferase assay using Bright-Glo™ Luciferase System (Promega) and Infinite F200 plate reader (Tecan) was used to read luminescence. Obtained relative light units (RLUs) were normalized to controls and dose-response curves were generated by nonlinear regression curve fitting with GraphPad Prism Version 8.0.2 (GraphPad Software, Inc., San Diego, CA) to calculate Neutralization Dose 50 (ND_50_).

### Functional repertoire analyses

nAbs VH and VL sequence reads were manually curated and retrieved using CLC sequence viewer (Qiagen). Aberrant sequences were removed from the data set. Analyzed reads were saved in FASTA format and the repertoire analyses were performed using Cloanalyst (http://www.bu.edu/computationalimmunology/research/software/)^48,49^.

### Statistical analysis

Statistical analysis was assessed with GraphPad Prism Version 8.0.2 (GraphPad Software, Inc., San Diego, CA). Nonparametric Mann–Whitney *t* test was used to evaluate statistical significance between the two groups analyzed in this study. Statistical significance was shown as * for values ≤0.05, ** for values ≤0.01, *** for values ≤0.001, and **** for values ≤0.0001.

### Reporting summary

Further information on research design is available in the Nature Research Reporting Summary linked to this article.

## Supplementary information

Supplementary Information

Peer Review File

Reporting Summary

## Data Availability

Source data are provided with this paper. All data supporting the findings in this study are available within the article or can be obtained from the corresponding author upon request. Source data are provided with this paper.

## Source data

Source Data

## References

[1] Asundi A, O’Leary C, Bhadelia N (2021). Global COVID-19 vaccine inequity: the scope, the impact, and the challenges. Cell Host Microbe.

[2] Kavanagh MM, Gostin LO, Sunder M (2021). Sharing technology and vaccine doses to address global vaccine inequity and end the COVID-19 pandemic. JAMA.

[3] Andreano E, Rappuoli R (2021). SARS-CoV-2 escaped natural immunity, raising questions about vaccines and therapies. Nat. Med..

[4] Burki T (2022). The origin of SARS-CoV-2 variants of concern. Lancet Infect. Dis..

[5] Callaway E (2021). Beyond Omicron: what’s next for COVID’s viral evolution. Nature.

[6] Karim SSA, Karim QA (2021). Omicron SARS-CoV-2 variant: a new chapter in the COVID-19 pandemic. Lancet.

[7] Viana, R. et al. Rapid epidemic expansion of the SARS-CoV-2 Omicron variant in southern Africa. *Nature* **603**, 679–686 (2022). 10.1038/s41586-022-04411-y PMC8942855 35042229

[8] Mykytyn, A. Z. et al. Omicron BA.1 and BA.2 are antigenically distinct SARS-CoV-2 variants. Preprint at *bioRxiv*10.1101/2022.02.23.481644 (2022).

[9] Chen, L.-L., Chu, A. W.-H., Zhang, R. R.-Q., Hung, I. F.-N. & To, K. K.-W. Serum neutralisation of the SARS-CoV-2 omicron sublineage BA.2. *Lancet Microbe* **3***,* E404 (2022). 10.1016/S2666-5247(22)00060-X PMC8959473 35373159

[10] Cameroni, E. et al. Broadly neutralizing antibodies overcome SARS-CoV-2 Omicron antigenic shift. *Nature* **602**, 664–670 (2022). 10.1038/s41586-021-04386-2 PMC9531318 35016195

[11] Planas, D. et al. Considerable escape of SARS-CoV-2 Omicron to antibody neutralization. *Nature* **602**, 671–675 (2022). 10.1038/s41586-021-04389-z 35016199

[12] Carreño JM (2022). Activity of convalescent and vaccine serum against SARS-CoV-2 Omicron. Nature.

[13] Hoffmann, M. et al. The Omicron variant is highly resistant against antibody-mediated neutralization: Implications for control of the COVID-19 pandemic. *Cell* **189**, 447–456.e11 (2022). 10.1016/j.cell.2021.12.032 PMC8702401 35026151

[14] McCallum M (2022). Structural basis of SARS-CoV-2 Omicron immune evasion and receptor engagement. Science.

[15] Cao, Y. et al. Omicron escapes the majority of existing SARS-CoV-2 neutralizing antibodies. *Nature* **602**, 657–663 (2022). 10.1038/s41586-021-04385-3 PMC8866119 35016194

[16] VanBlargan, L. A. et al. An infectious SARS-CoV-2 B.1.1.529 Omicron virus escapes neutralization by therapeutic monoclonal antibodies. *Nat. Med.* **28**, 490–495 (2022). 10.1038/s41591-021-01678-y PMC8767531 35046573

[17] Gruell, H. et al. mRNA booster immunization elicits potent neutralizing serum activity against the SARS-CoV-2 Omicron variant. *Nat. Med.* **28**, 477–480 (2022). 10.1038/s41591-021-01676-0 PMC8767537 35046572

[18] Dejnirattisai, W. et al. SARS-CoV-2 Omicron-B.1.1.529 leads to widespread escape from neutralizing antibody responses. *Cell* **185**, 467–484.e15 (2022). 10.1016/j.cell.2021.12.046 PMC8723827 35081335

[19] Yu, J. et al. Neutralization of the SARS-CoV-2 Omicron BA.1 and BA.2 variants. *N. Engl. J. Med.* **386**, 1579–1580 (2022). 10.1056/NEJMc2201849 PMC9006770 35294809

[20] Bruel, T. et al. Serum neutralization of SARS-CoV-2 Omicron sublineages BA.1 and BA.2 in patients receiving monoclonal antibodies. *Nat. Med.*10.1038/s41591-022-01792-5 (2022). 10.1038/s41591-022-01792-5 35322239

[21] Bowen, J. E. et al. Omicron BA.1 and BA.2 neutralizing activity elicited by a comprehensive panel of human vaccines. Preprint at *bioRxiv*10.1101/2022.03.15.484542 (2022).

[22] Andreano E (2021). Hybrid immunity improves B cells and antibodies against SARS-CoV-2 variants. Nature.

[23] Yi C (2020). Key residues of the receptor binding motif in the spike protein of SARS-CoV-2 that interact with ACE2 and neutralizing antibodies. Cell. Mol. Immunol..

[24] Torres, J. L. et al. Structural insights of a highly potent pan-neutralizing SARS-CoV-2 human monoclonal antibody. *Proc. Natl. Acad. Sci.*10.1073/pnas.2120976119 (2022). 10.1073/pnas.2120976119 PMC9171815 35549549

[25] Andreano E (2021). Extremely potent human monoclonal antibodies from COVID-19 convalescent patients. Cell.

[26] Barnes CO (2020). SARS-CoV-2 neutralizing antibody structures inform therapeutic strategies. Nature.

[27] Dejnirattisai W (2021). The antigenic anatomy of SARS-CoV-2 receptor binding domain. Cell.

[28] Pinto D (2020). Cross-neutralization of SARS-CoV-2 by a human monoclonal SARS-CoV antibody. Nature.

[29] Yuan M (2020). A highly conserved cryptic epitope in the receptor binding domains of SARS-CoV-2 and SARS-CoV. Science.

[30] Conforti A (2022). COVID-eVax, an electroporated DNA vaccine candidate encoding the SARS-CoV-2 RBD, elicits protective responses in animal models. Mol. Ther.: J. Am. Soc. Gene Ther..

[31] Voss William N (2021). Prevalent, protective, and convergent IgG recognition of SARS-CoV-2 non-RBD spike epitopes. Science.

[32] Crotty S (2021). Hybrid immunity. Science.

[33] Vanshylla K (2022). Discovery of ultrapotent broadly neutralizing antibodies from SARS-CoV-2 elite neutralizers. Cell host microbe.

[34] Yuan M (2020). Structural basis of a shared antibody response to SARS-CoV-2. Science.

[35] Greaney AJ (2021). Mapping mutations to the SARS-CoV-2 RBD that escape binding by different classes of antibodies. Nat. Commun..

[36] Turner JS (2021). SARS-CoV-2 mRNA vaccines induce persistent human germinal centre responses. Nature.

[37] Laidlaw BJ, Ellebedy AH (2022). The germinal centre B cell response to SARS-CoV-2. Nat. Rev. Immunol..

[38] Amanat F (2021). SARS-CoV-2 mRNA vaccination induces functionally diverse antibodies to NTD, RBD, and S2. Cell.

[39] Pajon, R. et al. SARS-CoV-2 Omicron variant neutralization after mRNA-1273 booster vaccination. *N. Engl. J. Med.* **386**, 1088–1091 (2022). 10.1056/NEJMc2119912 PMC8809504 35081298

[40] Hastie Kathryn M (2021). Defining variant-resistant epitopes targeted by SARS-CoV-2 antibodies: A global consortium study. Science.

[41] Tan C-W (2021). Pan-Sarbecovirus Neutralizing Antibodies in BNT162b2-Immunized SARS-CoV-1 Survivors. N. Engl. J. Med..

[42] Martinez David, R. et al. A broadly cross-reactive antibody neutralizes and protects against sarbecovirus challenge in mice. *Sci. Transl. Med.* **14**, eabj7125 (2022). 10.1126/scitranslmed.abj7125 PMC8899823 34726473

[43] Tortorici MA (2021). Broad sarbecovirus neutralization by a human monoclonal antibody. Nature.

[44] Wang Z (2021). Naturally enhanced neutralizing breadth against SARS-CoV-2 one year after infection. Nature.

[45] Tiller T (2008). Efficient generation of monoclonal antibodies from single human B cells by single cell RT-PCR and expression vector cloning. J. Immunol. Methods.

[46] Wardemann H, Busse CE (2019). Expression cloning of antibodies from single human B cells. Methods Mol. Biol..

[47] Notarbartolo S (2021). Integrated longitudinal immunophenotypic, transcriptional, and repertoire analyses delineate immune responses in patients with COVID-19. Sci. Immunol..

[48] Kepler TB (2013). Reconstructing a B-cell clonal lineage. I. Statistical inference of unobserved ancestors. F1000Res.

[49] Kepler TB (2014). Reconstructing a B-cell clonal lineage. II. Mutation, selection, and affinity maturation. Front. Immunol..

