# Supplementary material for: Anatomy of Omicron BA.1 and BA.2 neutralizing antibodies in COVID-19 mRNA vaccinees
Source: Nat Commun. 2022 Jun 13;13:3375. doi: 10.1038/s41467-022-31115-8 (PMC9189263; doi:10.1038/s41467-022-31115-8)
Supplement: Supplementary file 1 — Supplementary Information [file 41467_2022_31115_MOESM1_ESM.pdf]

Supplementary Information

Anatomy of Omicron BA.1 and BA.2 neutralizing antibodies in COVID-19 mRNA vaccinees

Emanuele Andreano[1,9], Ida Paciello[1,9], Silvia Marchese[2], Lorena Donnici[3], Giulio Pierleoni[4,5], Giulia Piccini[4], Noemi Manganaro[1], Elisa Pantano[1], Valentina Abbiento[1], Piero Pileri[1], Linda Benincasa[5], Ginevra Giglioli[5], Margherita Leonardi[4,5], Piet Maes[6], Concetta De Santi[1], Claudia Sala[1], Emanuele Montomoli[4,5,7], Raffaele De Francesco[2,3], Rino Rappuoli[1,8,*]

[1]Monoclonal Antibody Discovery (MAD) Lab, Fondazione Toscana Life Sciences, Siena, Italy

[2]Department of Pharmacological and Biomolecular Sciences DiSFeB, University of Milan, Milan, Italy

[3]INGM, Istituto Nazionale Genetica Molecolare "Romeo ed Enrica Invernizzi", Milan, Italy

[4]VisMederi S.r.l, Siena, Italy

[5]VisMederi Research S.r.l., Siena, Italy

[6]KU Leuven, Rega Institute, Department of Microbiology, Immunology and Transplantation, Laboratory of Clinical and Epidemiological Virology, Leuven, Belgium

[7]Department of Molecular and Developmental Medicine, University of Siena, Siena, Italy

[8]Department of Biotechnology, Chemistry and Pharmacy, University of Siena, Siena, Italy

[9]These authors contributed equally: Emanuele Andreano, Ida Paciello

*Corresponding author: Rino Rappuoli rino.r.rappuoli@gsk.com

SUPPLEMENTARY FIGURES

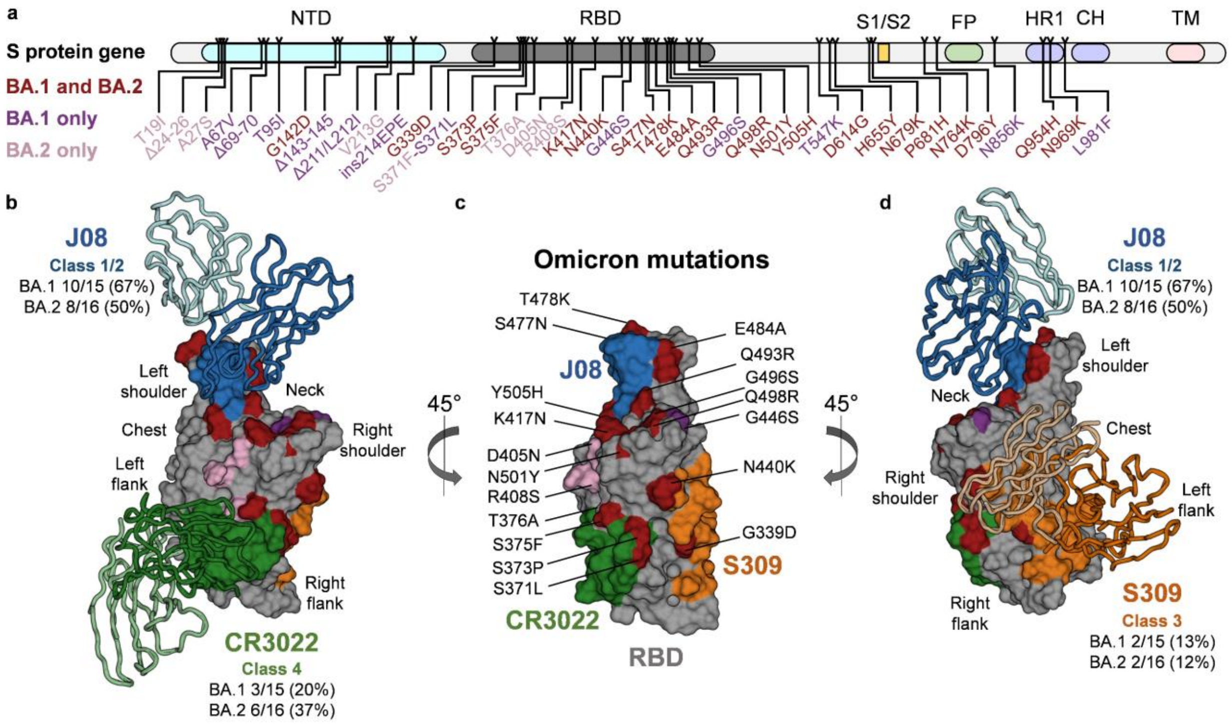

Supplementary Fig. 1. Distribution of BA.1 and BA.2 mutations on SARS-CoV-2 RBD. a, The graph shows the Omicron BA.1 and BA.2 S gene mutations based on the viral strain GISAID accession ID: EPI_ISL_6794907 (BA.1) and EPI_ISL_10654979 (BA.2). NTD, RBD, S1/S2 cleavage site, fusion peptide (FP), heptad repeat 1 (HP1), center helix (CH) and transmembrane domain (TM), are shown in light cyan, dark gray, yellow, light green, light violet and light pink respectively. **b-d**, Central panel shows the RBD harboring all 15 and 16 Omicron mutations for BA.1 and BA.2 respectively. Shared mutations are highlighted in dark red, while BA.1 and BA.2 specific mutations are shown in violet and pink respectively. J08 (representing Class 1/2 nAbs), S309 (representing Class 3 nAbs) and CR3022 (representing Class 4 nAbs) epitopes are highlighted in light blue, orange and green respectively (**c**). Left panel shows RBD rotated by 45 degrees on the right and the distribution of Omicron BA.1 and BA.2 mutations on Class 1/2 and Class 4 epitope regions (**b**). Right panel shows RBD rotated by 45 degrees on the left and the distribution of Omicron BA.1 and BA.2 mutations on Class 1/2 and Class 3 epitope regions (**d**).

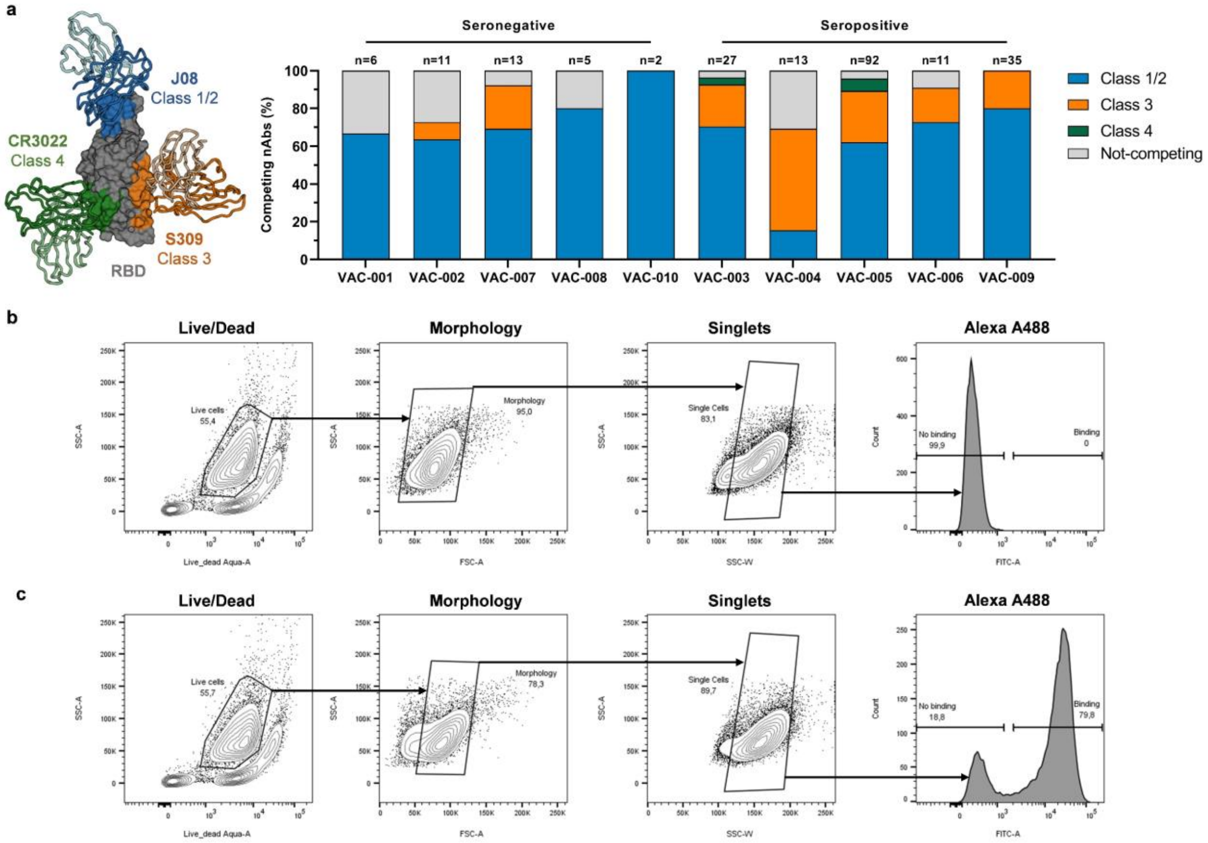

Supplementary Fig. 2. nAbs distribution to SARS-CoV-2 RBD and binding to SARS-CoV-1 spike protein. a, Left panel shows nAbs binding regions on the RBD. Right panel shows a bar graph for competition assay. Class 1/2, Class 3, Class 4 and Not-competing nAbs are shown in light blue, orange, green and light gray respectively. The number (n) of antibodies tested per each donor is denoted on the graph. (**b-c**) The gating strategy shows from left to right: Live/Dead, morphology, singlets and Goat anti-Human IgG Secondary Antibody-Alexa Fluor 488 for the negative (secondary antibody alone) (**a**) and positive (SARS1 antibody binder S309) (**b**) controls respectively. Percentages of single populations and boundaries between positive and negative cells are denoted on each graph.

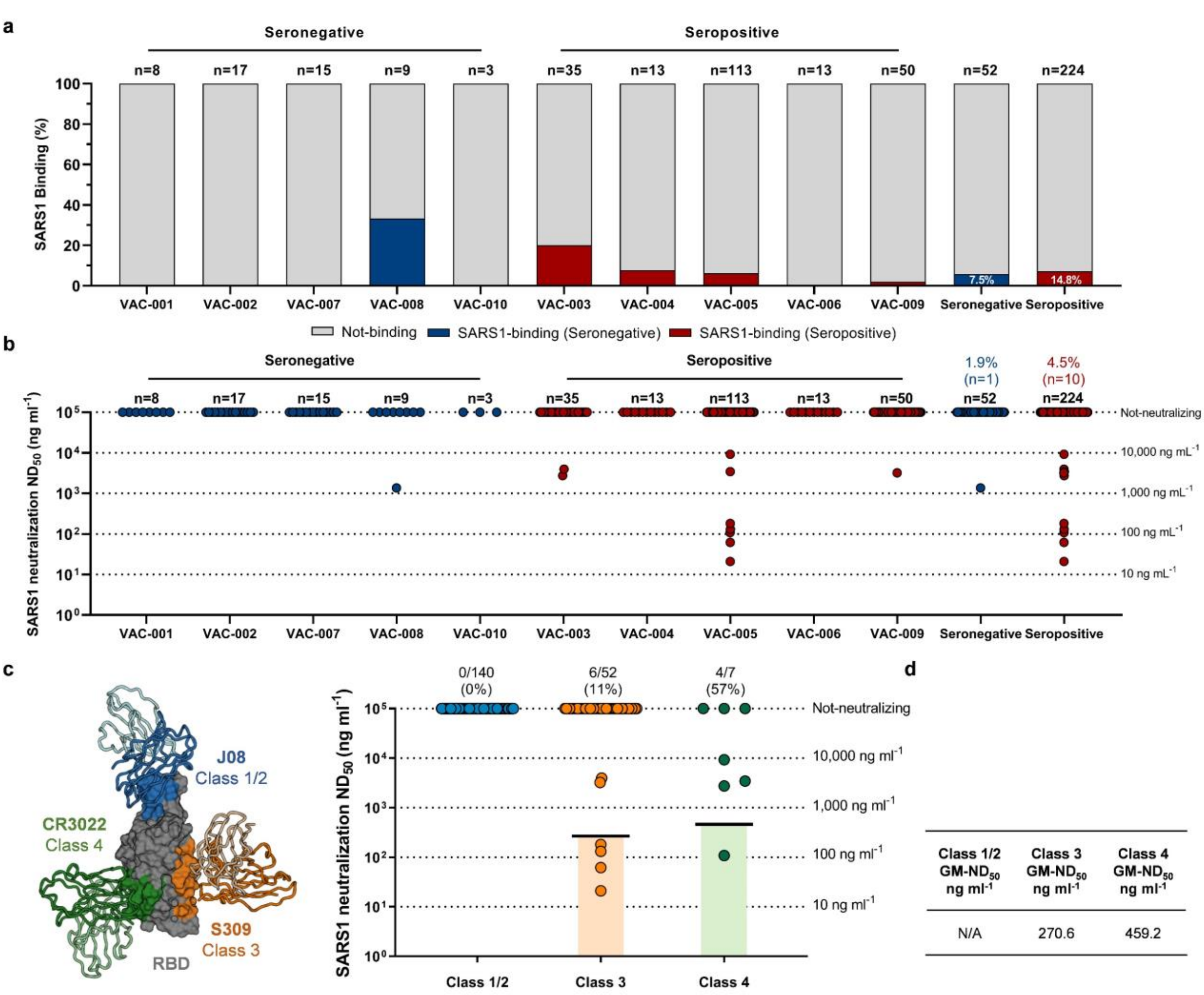

Class 1/2 GM-ND50 ng ml-1	Class 3 GM-ND50 ng ml-1	Class 4 GM-ND50 ng ml-1
N/A	270.6	459.2

Supplementary Fig. 3. Functional characterization of SARS1 nAbs. a, The bar graph shows the percentage of not-binding antibodies (grey), SARS1-binding nAbs from individuals who were seronegative (dark blue) and SARS1-binding nAbs for individuals who were seropositive (dark red). The total number (n) of antibodies tested per individual is shown on the top of each bar. **b**, Dot chart shows the neutralization potency, reported as ND_{50} (ng ml^{-1}), of nAbs isolated from seronegatives and seropositives against SARS1. The number and percentage of nAbs from individuals who were seronegative and seropositive and neutralization ND_{50} (ng ml^{-1}) ranges (black dotted lines) are denoted on the graph. **c-d**, dot chart shows Class 1/2, Class 3 and Class4 distribution of SARS1 nAbs (**c**). Neutralization potencies, reported as ND_{50} (ng ml^{-1}), and geometric mean (black line) are denoted on the graph. The table shows the ND_{50} (ng ml^{-1}) geometric mean (GM) of all nAbs pulled together from each group against SARS1. Technical triplicates were performed for each experiment (**d**).

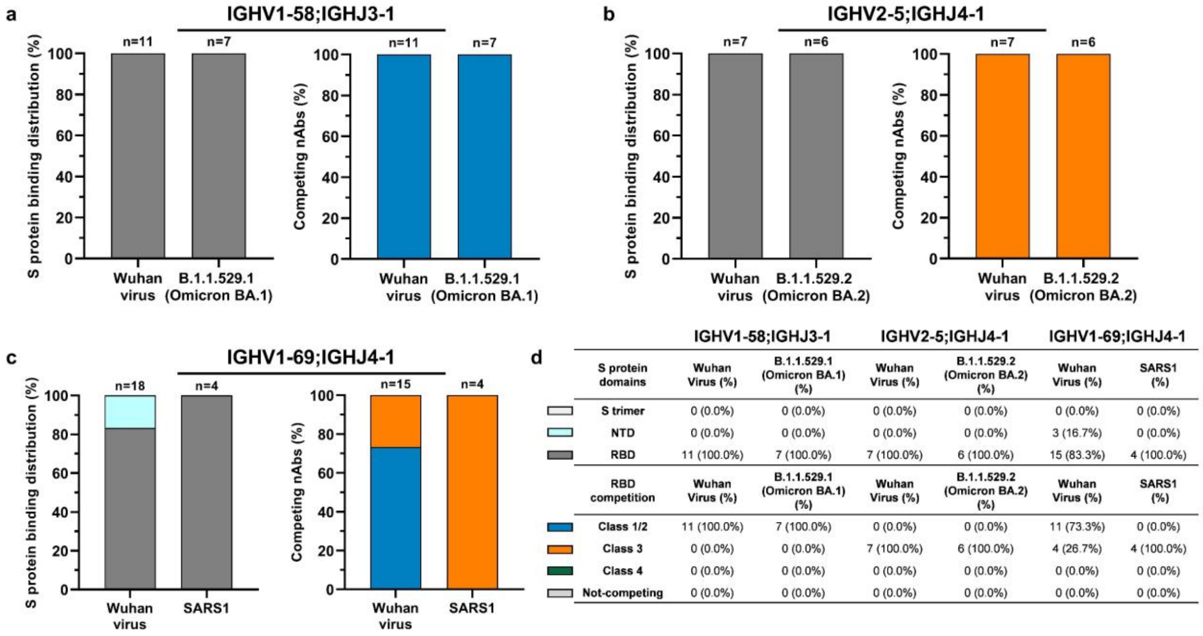

	IGHV1-58;IGHJ3-1		IGHV2-5;IGHJ4-1		IGHV1-69;IGHJ4-1	
S protein domains	Wuhan Virus (%)	B.1.1.529.1 (Omicron BA.1) (%)	Wuhan Virus (%)	B.1.1.529.2 (Omicron BA.2) (%)	Wuhan Virus (%)	SARS1 (%)
S trimer	0 (0.0%)	0 (0.0%)	0 (0.0%)	0 (0.0%)	0 (0.0%)	0 (0.0%)
NTD	0 (0.0%)	0 (0.0%)	0 (0.0%)	0 (0.0%)	3 (16.7%)	0 (0.0%)
RBD	11 (100.0%)	7 (100.0%)	7 (100.0%)	6 (100.0%)	15 (83.3%)	4 (100.0%)
RBD competition	Wuhan Virus (%)	B.1.1.529.1 (Omicron BA.1) (%)	Wuhan Virus (%)	B.1.1.529.2 (Omicron BA.2) (%)	Wuhan Virus (%)	SARS1 (%)
Class 1/2	11 (100.0%)	7 (100.0%)	0 (0.0%)	0 (0.0%)	11 (73.3%)	0 (0.0%)
Class 3	0 (0.0%)	0 (0.0%)	7 (100.0%)	6 (100.0%)	4 (26.7%)	4 (100.0%)
Class 4	0 (0.0%)	0 (0.0%)	0 (0.0%)	0 (0.0%)	0 (0.0%)	0 (0.0%)
Not-competing	0 (0.0%)	0 (0.0%)	0 (0.0%)	0 (0.0%)	0 (0.0%)	0 (0.0%)

Supplementary Fig. 4. Binding distribution of BA.1, BA.2 and SARS1 predominant nAbs. a-c, Bar graphs show the percentages of IGHV1-58;IGHJ3-1 (**a**), IGHV2-5;IGHJ4-1 (**b**) and IGHV1-69;IGHJ4-1 (**c**) nAbs binding to the S protein trimer (light gray), NTD (cyan) and RBD (dark gray), and targeting Class 1/2 (blue), Class 3 (orange) and Class 4 (dark green) epitope regions on the RBD as well as and Not-competing nAbs (gray). Number of antibodies per group are denoted on each bar. **d**, the table summarizes number and percentages of S protein trimer, NTD and RBD binding nAbs, and Class 1/2, Class 3, Class 4 and not-competing nAbs.

SUPPLEMENTARY TABLES

Supplementary Table 1. Summary of BA.1, BA.2 and SARS1 antibodies

Subject	SARS-CoV-2 serology	Wuhan - Neutralizing antibodies (n)	BA.1 - Neutralizing antibodies (n)	BA.1 - Neutralizing antibodies (%)	BA.2 - Neutralizing antibodies (n)	BA.2 - Neutralizing antibodies (%)	SARS1 - Binding antibodies (n)	SARS1 - Binding antibodies (%)	SARS1 - Neutralizing antibodies (n)	SARS1 - Neutralizing antibodies (%)
**VAC-001**	Seronegative	8	0	0.0	0	0.0	0	0.0	0	0.0
**VAC-002**	Seronegative	17	0	0.0	1	5.9	0	0.0	0	0.0
**VAC-007**	Seronegative	15	1	6.7	2	13.3	0	0.0	0	0.0
**VAC-008**	Seronegative	9	0	0.0	1	11.1	3	33.3	1	11.1
**VAC-010**	Seronegative	3	0	0.0	0	0.0	0	0.0	0	0.0
**Total (Seronegative)**		**52**	**1**	**1.9**	**4**	**7.7**	**3**	**5.8**	**1**	**1.9**
**VAC-003**	Seropositive	35	5	14.3	3	8.6	7	20.0	2	5.7
**VAC-004**	Seropositive	13	2	15.4	3	23.1	1	7.7	0	0.0
**VAC-005**	Seropositive	113	24	21.2	35	31.0	7	6.2	7	6.2
**VAC-006**	Seropositive	13	2	15.4	3	23.1	0	0.0	0	0.0
**VAC-009**	Seropositive	50	5	10.0	7	14.0	1	2.0	1	2.0
**Total (Seropositive)**		**224**	**38**	**16.9**	**51**	**22.8**	**16**	**7.1**	**10**	**4.5**

Supplementary Table 2. Competition assay summary

Subject	SARS-CoV-2 serology	Distribution Competition Class 1/2 (n)	Distribution Competition Class 1/2 (%)	Distribution Competition Class 3 (n)	Distribution Competition Class 3 (%)	Distribution Competition Class 4 (n)	Distribution Competition Class 4 (%)	Distribution Competition Not-competing (n)	Distribution Competition Not-competing (%)
**VAC-001**	Seronegative	4	10.8	0	0.0	0	0.0	2	5.4
**VAC-002**	Seronegative	7	18.9	1	2.7	0	0.0	3	8.1
**VAC-007**	Seronegative	9	24.3	3	8.1	0	0.0	1	2.7
**VAC-008**	Seronegative	4	10.8	0	0.0	0	0.0	1	2.7
**VAC-010**	Seronegative	2	5.4	0	0.0	0	0.0	0	0.0
**Total (Seronegative)**		**26**	**70.3**	**4**	**10.8**	**0**	**0.0**	**7**	**18.9**
**VAC-003**	Seropositive	19	10.7	6	3.9	1	0.5	1	0.6
**VAC-004**	Seropositive	2	1.1	7	3.9	0	0.0	4	2.2
**VAC-005**	Seropositive	57	32.0	25	14.0	6	3.4	4	2.2
**VAC-006**	Seropositive	8	4.5	2	1.1	0	0.0	1	0.6
**VAC-009**	Seropositive	28	15.7	7	3.9	0	0.0	0	0.0
**Total (Seropositive)**		**114**	**64.0**	**47**	**26.9**	**7**	**3.9**	**10**	**5.6**
