# Supplementary material for: Anatomy of Omicron BA.1 and BA.2 neutralizing antibodies in COVID-19 mRNA vaccinees
Source: Nat Commun. 2022 Jun 13;13:3375. doi: 10.1038/s41467-022-31115-8 (PMC9189263; doi:10.1038/s41467-022-31115-8)
Supplement: Supplementary file 3 — Reporting Summary [file 41467_2022_31115_MOESM3_ESM.pdf]

nature portfolio

Corresponding author(s): Rino Rappuoli

Last updated by author(s): May 20, 2022

Reporting Summary

Nature Portfolio wishes to improve the reproducibility of the work that we publish. This form provides structure for consistency and transparency in reporting. For further information on Nature Portfolio policies, see our Editorial Policies and the Editorial Policy Checklist.

Statistics

For all statistical analyses, confirm that the following items are present in the figure legend, table legend, main text, or Methods section.

n/a	Confirmed	
☐	☒	The exact sample size (*n*) for each experimental group/condition, given as a discrete number and unit of measurement
☐	☒	A statement on whether measurements were taken from distinct samples or whether the same sample was measured repeatedly
☐	☒	The statistical test(s) used AND whether they are one- or two-sided<br>*Only common tests should be described solely by name; describe more complex techniques in the Methods section.*
☒	☐	A description of all covariates tested
☒	☐	A description of any assumptions or corrections, such as tests of normality and adjustment for multiple comparisons
☐	☒	A full description of the statistical parameters including central tendency (e.g. means) or other basic estimates (e.g. regression coefficient) AND variation (e.g. standard deviation) or associated estimates of uncertainty (e.g. confidence intervals)
☐	☒	For null hypothesis testing, the test statistic (e.g. *F*, *t*, *r*) with confidence intervals, effect sizes, degrees of freedom and *P* value noted<br>*Give P values as exact values whenever suitable.*
☒	☐	For Bayesian analysis, information on the choice of priors and Markov chain Monte Carlo settings
☒	☐	For hierarchical and complex designs, identification of the appropriate level for tests and full reporting of outcomes
☒	☐	Estimates of effect sizes (e.g. Cohen's *d*, Pearson's *r*), indicating how they were calculated

Our web collection on statistics for biologists contains articles on many of the points above.

Software and code

Policy information about availability of computer code

Data collection	- BD Biosciences BD FACSDiva Software v9.0
Data analysis	- GraphPad Prism 8.0.2 was used to perform statistical analyses<br>- BD FlowJo 10.5.3<br>- Boston University, Cloanalyst (http://www.bu.edu/computationalimmunology/research/software/) 0.9.0.0

For manuscripts utilizing custom algorithms or software that are central to the research but not yet described in published literature, software must be made available to editors and reviewers. We strongly encourage code deposition in a community repository (e.g. GitHub). See the Nature Portfolio guidelines for submitting code & software for further information.

Data

Policy information about availability of data

All manuscripts must include a data availability statement. This statement should provide the following information, where applicable:

- Accession codes, unique identifiers, or web links for publicly available datasets
- A description of any restrictions on data availability
- For clinical datasets or third party data, please ensure that the statement adheres to our policy

Source data are provided with this paper. All data supporting the findings in this study are available within the article or can be obtained from the corresponding author upon request.

Field-specific reporting

Please select the one below that is the best fit for your research. If you are not sure, read the appropriate sections before making your selection.

- [x] Life sciences
- [ ] Behavioural & social sciences
- [ ] Ecological, evolutionary & environmental sciences

For a reference copy of the document with all sections, see nature.com/documents/nr-reporting-summary-flat.pdf

Life sciences study design

All studies must disclose on these points even when the disclosure is negative.

Sample size	We analyzed of 276 human neutralizing antibodies isolated from 10 previously enrolled COVID-19 vaccinated subjects (5 seronegative and 5 seropositive), to evaluate cross-protection of vaccine induced-antibodies against SARS-CoV-2 B.1.1.529.1 (BA.1), B.1.1.529.2 (BA.2), and SARS-CoV-1. Given the exploratory nature of the study, we did not use statistical methods to predetermine sample size of human monoclonal antibodies tested per each group. Sample size was based on previous studies that applied a similar technology (https://doi.org/10.1038/s41586-021-04117-7; https://doi.org/10.15252/emmm.202114035). The authors believed that by analyzing the neutralizing antibody response of 5 subjects/group were a good balance between feasibility of analyzing at single cell level several thousands of memory B cells and the ability to represent the antibody response of seronegative and seropositive people.
Data exclusions	No data was excluded.
Replication	All experiments were performed in technical duplicates or triplicates as indicated in the figure legends and methods section.
Randomization	The experiments were not randomized and all available samples were tested. The authors aimed to specifically assess the antibody response of seronegative and seropositve subjects. Based on what stated above, the authors believed that randomization was not appropriate.
Blinding	The investigators were not blinded during group allocation, data collection and analyses.

Reporting for specific materials, systems and methods

We require information from authors about some types of materials, experimental systems and methods used in many studies. Here, indicate whether each material, system or method listed is relevant to your study. If you are not sure if a list item applies to your research, read the appropriate section before selecting a response.

Materials & experimental systems

n/a	Involved in the study	
[ ]	[x]	Antibodies
[ ]	[x]	Eukaryotic cell lines
[x]	[ ]	Palaeontology and archaeology
[x]	[ ]	Animals and other organisms
[x]	[ ]	Human research participants
[x]	[ ]	Clinical data
[x]	[ ]	Dual use research of concern

Methods

n/a	Involved in the study	
[x]	[ ]	ChIP-seq
[ ]	[x]	Flow cytometry
[x]	[ ]	MRI-based neuroimaging

Antibodies

Antibodies used	Invitrogen (Thermo Fisher) Goat anti-Human IgG (H+L) Cross-Adsorbed Secondary Antibody, Alexa Fluor 488, Cat#A11013, polyclonal, Lot#2273669
Validation	Invitrogen (Thermo Fisher) Goat anti-Human IgG (H+L) Cross-Adsorbed Secondary Antibody, Alexa Fluor 488, polyclonal, QC testing, reactivity human, application flow cytometry (https://www.thermofisher.com/order/genome-database/dataSheetPdf?producttype=antibody&productsubtype=antibody_secondary&productId=A-11013&version=205)

Eukaryotic cell lines

Policy information about cell lines

Cell line source(s)	VERO E6 cell line ATCC Cat#CRL-1586; Expi293F cells Thermo Fisher Cat#A14527
Authentication	These cell lines were obtained from vendors that sell authenticated cell lines, they grew, performed and showed morphology as expected. No additional specific authentication was performed.
Mycoplasma contamination	Vero E6 cell lines are routinely tested on a monthly basis and tested negative for mycoplasma. Expi293F cells were not tested

	for mycoplasma contamination.
Commonly misidentified lines (See ICLAC register)	No commonly misidentified cell lines were used in this study.

Flow Cytometry

Plots

Confirm that:

- [x] The axis labels state the marker and fluorochrome used (e.g. CD4-FITC).
- [x] The axis scales are clearly visible. Include numbers along axes only for bottom left plot of group (a 'group' is an analysis of identical markers).
- [x] All plots are contour plots with outliers or pseudocolor plots.
- [x] A numerical value for number of cells or percentage (with statistics) is provided.

Methodology

Sample preparation	Expi293F cells (Thermo Fisher) were transiently transfected with SARS-CoV-1 S-protein expression vectors (pcDNA3.3_CoV1_D28) using Expifectamine Enhancer according to the manufacturer's protocol (Thermo Fisher). Two days later, to exclude dead cells from analysis, Expi293F were harvested, dispensed into a 96-well plate (3x10^5 cell/well), and stained for 30 minutes at room temperature (RT) with Live/Dead Fixable Aqua reagent (Invitrogen; Thermo Scientific) diluted 1:500. Following Live/Dead staining, cells were washed with PBS and incubated with nAbs candidates for 40 minutes at RT. Next, to identify the SARS-CoV-1 S protein mAbs binders, cells were washed and stained with the Alexa Fluor 488-labelled secondary antibody Goat anti-Human IgG (H+L) secondary antibody (Invitrogen) diluted 1:500. After 40 minutes of incubation, labeled cells were washed, resuspended in 150 µL of PBS and analyzed using the BD LSR II flow cytometer (Becton Dickinson). Cells incubated with the SARS-CoV-1 nAb binder (S309) or incubated only with the secondary antibody were used as positive and negative controls respectively. Data were analyzed with FlowJo™ Software (version 10).
Instrument	BD LSR II flow cytometer BD Biosciences
Software	BD Biosciences BD FACSDiva Software v9.0
Cell population abundance	The binding ability to SARS-CoV-1 S protein of tested monoclonal antibodies ranged from 0 to 86.95% of transfected Expi293F cells. Gating strategy for SARS-CoV-1 S protein binding antibodies was based on the negative control as reported in the Extended Data (Extended Data Figure 1a,b).
Gating strategy	The gating strategy used to identify SARS-CoV-1 S protein binding antibodies is shown in the Extended Data (Extended Data Figure 2 b-c). Cells were gated for Live/dead, morphology singlets and percentages of positive/negative Alexa488 signal was evaluated. Boundaries between "positive" and "negative" cells are defined and denoted on each graph.

- [x] Tick this box to confirm that a figure exemplifying the gating strategy is provided in the Supplementary Information.
